# Exome Analyses of Long QT Syndrome Reveal Candidate Pathogenic Mutations in Calmodulin-Interacting Genes

**DOI:** 10.1371/journal.pone.0130329

**Published:** 2015-07-01

**Authors:** Daichi Shigemizu, Takeshi Aiba, Hidewaki Nakagawa, Kouichi Ozaki, Fuyuki Miya, Wataru Satake, Tatsushi Toda, Yoshihiro Miyamoto, Akihiro Fujimoto, Yutaka Suzuki, Michiaki Kubo, Tatsuhiko Tsunoda, Wataru Shimizu, Toshihiro Tanaka

**Affiliations:** 1 Laboratory for Medical Science Mathematics, RIKEN Center for Integrative Medical Sciences, Yokohama, Japan; 2 Department of Cardiovascular Medicine, National Cerebral and Cardiovascular Center, Suita, Japan; 3 Laboratory for Genome Sequencing Analysis, RIKEN Center for Integrative Medical Sciences, Yokohama, Japan; 4 Laboratory for Cardiovascular Diseases, RIKEN Center for Integrative Medical Sciences, Yokohama, Japan; 5 Division of Neurology/Molecular Brain Science, Kobe University Graduate School of Medicine, Kobe, Japan; 6 Department of Preventive Cardiology, National Cerebral and Cardiovascular Center, Suita, Japan; 7 Department of Computational Biology, Division of Biosystem Sciences, University of Tokyo, Chiba, Japan; 8 Laboratory for Genotyping Development, RIKEN Center for Integrative Medical Sciences, Yokohama, Japan; 9 Department of Cardiovascular Medicine, Nippon Medical School, Tokyo, Japan; 10 Department of Human Genetics and Disease Diversity, Tokyo Medical and Dental University Graduate School of Medical and Dental Sciences, Tokyo, Japan; CNR, ITALY

## Abstract

Long QT syndrome (LQTS) is an arrhythmogenic disorder that can lead to sudden death. To date, mutations in 15 LQTS-susceptibility genes have been implicated. However, the genetic cause for approximately 20% of LQTS patients remains elusive. Here, we performed whole-exome sequencing analyses on 59 LQTS and 61 unaffected individuals in 35 families and 138 unrelated LQTS cases, after genetic screening of known LQTS genes. Our systematic analysis of familial cases and subsequent verification by Sanger sequencing identified 92 candidate mutations in 88 genes for 23 of the 35 families (65.7%): these included eleven *de novo*, five recessive (two homozygous and three compound heterozygous) and seventy-three dominant mutations. Although no novel commonly mutated gene was identified other than known LQTS genes, protein-protein interaction (PPI) network analyses revealed ten new pathogenic candidates that directly or indirectly interact with proteins encoded by known LQTS genes. Furthermore, candidate gene based association studies using an independent set of 138 unrelated LQTS cases and 587 controls identified an additional novel candidate. Together, mutations in these new candidates and known genes explained 37.1% of the LQTS families (13 in 35). Moreover, half of the newly identified candidates directly interact with calmodulin (5 in 11; comparison with all genes; p=0.042). Subsequent variant analysis in the independent set of 138 cases identified 16 variants in the 11 genes, of which 14 were in calmodulin-interacting genes (87.5%). These results suggest an important role of calmodulin and its interacting proteins in the pathogenesis of LQTS.

## Introduction

Long QT syndrome (LQTS) is characterized by a prolonged QT interval in the electrocardiogram (ECG) and ventricular tachyarrhythmia. Arrhythmia is often triggered by exercise, particularly swimming, or emotional stress, resulting in recurrent syncope, seizures, and sometimes, sudden, unexpected cardiac death [1].

LQTS has an estimated prevalence as high as one in 2,000 people [2]. To date, mutations in 15 susceptibility genes have been identified. The majority of those affected have mutations in *KCNQ1* (LQT1), *KCNH2* (LQT2) and *SCN5A* (LQT3), encoding potassium and sodium ion channel alpha-subunits. These three genes account for 75% of LQTS cases (LQT1: 30%-35%, LQT2: 25%-30%, LQT3: 5%-10%), while the remaining known LQTS genes, which encode beta subunits of plasma membrane channels, channel-interacting proteins, structural membrane scaffolding proteins or membrane anchoring proteins, account for only 5% of cases [3]. Mutations have not been detected in the remaining 20% of patients.

Whole-exome sequencing (WES) is widely used to identify genetic variations in coding regions [4]. WES is more powerful and cost-effective for exonic regions than whole-genome sequencing because it obtains a deeper coverage of the target regions. WES has been recently used to successfully identify causal mutations of Mendelian diseases [5, 6] and driver mutations in tumors [7–9].

Here, we report the identification of candidate pathogenic mutations, through WES and validated by Sanger sequencing, in two-thirds of the examined LQTS families. Although no commonly mutated gene was identified other than known genes, protein-protein interaction (PPI) network analysis revealed that ten candidates interact with proteins encoded by known LQTS genes. Interestingly, half of these directly interact with calmodulin, which is statistically significant when compared to the number of molecules that directly interact with calmodulin. In addition, candidate gene based association studies using an independent set of unrelated LQTS individuals and unaffected individuals identified an additional novel LQTS candidate. Examination of the presence of mutations in these candidate genes in the unrelated LQTS cases revealed that most mutations were in calmodulin-interacting genes. We believe these findings contribute to a greater understanding of LQTS and provide clues for future research into its pathogenic mechanism.

## Materials and Methods

### Ethics Statement

This study was approved by the ethics committee of the Institutes of National Cerebral and Cardiovascular Center and RIKEN. The design and performance of the current study involving human subjects were clearly described in a research protocol. All participants provided written informed consent before taking part in this research.

### Study subjects

LQTS is diagnosed using the following criteria: patients with a Schwartz risk score > 3.5 in the absence of a secondary cause for QT prolongation [10], and/or an unequivocally pathogenic mutation in one of the LQTS genes, or QTc > 500 ms in repeated 12-lead ECG in the absence of a secondary cause for QT prolongation. Among the LQTS patients registered at National Cerebral and Cardiovascular Center who provided written informed consent, we recruited 186 genetically unrelated LQTS cases whose mutations were not detected by genetic screening of known LQTS genes. Among them, 35 had family data (21 family trio data of LQTS patients with unaffected parents and 14 pedigrees with at least one additional LQTS family member, S1 Fig) with DNA samples, and therefore were selected for pedigree analysis. Therefore, among the 35 families, excluding the proband, an additional 85 family members (24 LQTS and 61 unaffected control subjects) were recruited for this analysis. We also included the remaining 151 samples with no family data as genetically independent LQTS cases. In total, 271 samples were subjected to WES analysis. In the course of the analysis, we detected mutations in known LQTS genes for 13 out of 151 non-pedigree cases (Table 1) [11–14] and these individuals were excluded from further analysis. Consequently, we examined 59 LQTS and 61 unaffected individuals in 35 families and 138 unrelated LQTS cases (n = 258). The participant summary, including gender, average age, and the other clinical information, is shown in Table 2.

**Table 1:** Identification of known-gene and disease-causing variant in the LQTS.

Phenotype	Gene symbol	Transcript ID	cDNA level change	Protein level change	QTc (ms)	Symptoms	HGMD^†^, others
LQT1*	*KCNQ1*	NM_000218.2	c.760G>A	p.V254M	570	syncope	CM960898 [11]
LQT1*	*KCNQ1*	NM_000218.2	c.965C>T	p.T322M	474	asympt	CM057152 [12]
LQT1	*KCNQ1*	NM_000218.2	c.683+2T>G	-	470	Asymp	Pedigree analysis
LQT1	*KCNQ1*	NM_000218.2	c.1032+1G>A	-	572	TdP VF Sym40yo	Pedigree analysis
LQT2*	*KCNH2*	NM_000238.3	c.1849T>C	p.F617L	475	asympt	
LQT2*	*KCNH2*	NM_000238.3	c.1831T>G	p.Y611D	490	VF	CM107399 [13]
LQT2*	*KCNH2*	NM_000238.3	c.307+2T>A	-	548	asympt	
LQT3*	*SCN5A*	NM_001160160.1	c.4900G>A	p.V1634I	448	TdP	
LQT4*	*ANK2*	NM_001148.4	c.2474C>T	p.T825I	436	syncope	
LQT4*	*ANK2*	NM_001148.4	c.4876A>G	p.K1626E	650	syncope	
LQT4*	*ANK2*	NM_001148.4	c.6149T>C	p.I2050T	464	VF	
LQT4*	*ANK2*	NM_001148.4	c.8123T>C	p.V2708A	420	asympt	
LQT5	*KCNE1*	NM_000219.3	c.253G>A	p.D85N	492	asympt	CM040436 [14], Pedigree analysis, rare variant
LQT9	*CAV3*	NM_033337.2	c.37A>T	p.I13F	466	asympt	Pedigree analysis, SNV
LQT11*	*AKAP9*	NM_147185.2	c.2295T>A	p.D765E	453	asympt	
LQT11*	*AKAP9*	NM_147185.2	c.5341T>A	p.S1781T	457	asympt	
LQT12*	*SNTA1*	NM_003098.2	c.1498C>T	p.R500C	444	asympt	
	*NOS1AP*	NM_014697.2	c.1276G>A	p.V426M	413	asympt	disease causing variant
	*NOS1AP*	NM_014697.2	c.824C>T	p.S275F	453	syncope	disease causing variant

asympt; asymptomatic, SNV: single nucleotide variant

^†^accession number obtained from HGMD professional (ver. 2014.4, accessed on Mar. 19, 2015)

*mutations detected in known LQTS genes for non-pedigree cases.

**Table 2:** Clinical background of LQTS patients and their family members.

	35 LQTS families		unrelated LQTS (n = 138)
	LQTS (n = 59)	Control (n = 61)	
age	23±18	25±18	19±16
male/ female	20/39	36/25	54/84
QTc (ms)	480±40	402±21	466±49
syncope, n (%)	24 (41)	1 (1)	59 (43)
VF or CA, n (%)	9 (15)	0 (0)	18 (13)

VF: ventricular fibrillation, CA: cardiac arrest

### Whole-exome sequencing

Exome capture was performed by the Agilient SureSelect Human All Exon V4 according to the manufacturer’s instructions. This kit captures genomic DNA by in-solution hybridization with RNA oligonucleotides, enabling specific targeting of approximately 51Mb of the human genome. The captured DNA was sequenced using the Illumina HiSeq2000 platform with paired-end reads of 101bp for insert libraries of 150–200bp according to the manufacturer’s instructions.

### Exome sequence data analysis

Read sequences were mapped by the Burrows-Wheeler Aligner (BWA: version 0.6.1) [15] to the human reference genome (GRCh37). Duplicate PCR reads were identified and removed using SAMtools (version 0.1.8) [16] and in-house software. After filtering by pair mapping distance, mapping uniqueness and pair orientation, the mapping result files were converted into pileup format using SAMtools. Variant calling was conducted based on methods we have published elsewhere, VCMM [17]. We used the following quality control filters: (i) alignments near putative indels were refined using GATK [18]; (ii) a stand bias filter excluded variants whose alternative allele was preferentially found in one of the two available read orientations at the site.

Variants that were found in dbSNP (version 137) [19], 1000 Genomes Project (n = 1,094) [20], NHLBI Exome Sequencing Project Exome Variant Server (n = 6,503; http://evs.gs.washington.edu/EVS/) [accessed June 2012] (ESP6500) [21] and our in-house whole genome and exome data composed of 1,257 non-cardiac Japanese individuals were excluded from further analyses. Nongenic, intronic and synonymous variants other than those occurring at canonical splice sites and non-synonymous variants predicted as benign/tolerant by both SIFT (http://sift.jcvi.org/www/) [22] and PolyPhen-2 (http://genetics.bwh.harvard.edu/pph2/) [23] were also excluded. Furthermore, we assumed that affected individuals had *de novo* or recessive (both homozygous and compound heterozygous) mutations for parent/affected offspring trio families and dominant for the other families. All candidate mutations were validated using Sanger sequencing of both the affected and unaffected individuals.

All mutations in known LQTS genes and in candidate genes, identified in this study, have been deposited into NCBI ClinVar with the accession numbers SCV000221974—SCV000222093.

### Network analysis

Network analysis was performed using the Ingenuity Pathway Analysis software (IPA; Ingenuity Systems) based on the 15 known LQTS genes and the 88 candidate pathogenic genes identified. We considered molecules and/or relationships available in the IPA Knowledge Base for human, mouse and rat and set the confidence filter to experimentally observed or high (predicted). Networks were generated with a maximum size of 35 genes and allowing up to 10 networks. Molecules in the query set with recorded interactions were eligible for network construction using the IPA algorithm. Networks were ranked by IPA network score according to their degree of relevance to the eligible molecules in the query data set. The network score is calculated using Fisher’s exact test on a basis of the number of eligible molecules in the network and its size, as well as the total number of eligible molecules analyzed and the total number of molecules in the Ingenuity Knowledge Base that could potentially be included in the networks.

### Quality control and gene-based association study

We used 748 Japanese individuals, which included 161 LQTS cases (23 probands in LQTS families and 138 independent LQTS patients) and 587 controls. Closely related subjects, where the identity-by-descent (IBD) proportion of alleles shared was over 0.125, and outliers by principal-component analysis (PCA) [24] (S2 Fig) were previously excluded. We estimated the IBD sharing score using PLINK’s ‘-genome’ option [25] and performed PCA using *gdsfmt* and *SNPRelate* packages in the statistical software R [26]. We also excluded all SNVs with a genotype call rate < 0.80, a Hardy-Weinberg equilibrium p-value < 1×10^-6^ or nongenic and intronic variants other than those occurring at canonical splice sites. When also considering a MAF < 0.005, 51,393 SNVs passed these stringent quality control criteria. The quantile-quantile (QQ) plots of the p-values from the Cochran-Armitage test for trend showed the genomic inflation factor *λ*_*GC*_ to be 1.027 (S3 Fig).

For the gene-based association studies, we used the SKAT-O test [27], which encompasses both burden tests (e.g. CMC method [28]) and variance-component tests (e.g. SKAT [29]). We performed the analysis using default weights and MAF < 0.01 for the combination of non-synonymous variants predicted to be damaging by SIFT [22] or PolyPhen-2 [23] analysis and splice-site variants. We performed the test for candidate genes with at least two variants and declared a gene-based test association significant when q-value < 0.05.

## Results

### Identification of candidate mutations in probands

**Fig 1:**
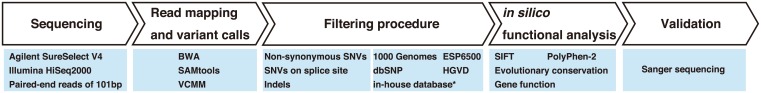
Experimental work flow for detecting sequence variants by WES. In-house database with asterisk is our in-house whole exome or whole genome data composed of 1,257 non-cardiac Japanese individuals.

On average, 6.7 Gbp of short read sequence data were obtained from WES (S1 Table). In total, 68.6% of the sequenced bases were mapped to the targeted regions and 92.8% of mapped exon sequences had at least ten times coverage (S4 Fig). The average coverage was 68X across individuals. An average of 19,505 coding SNVs and 516 coding insertion/deletion (indels) were identified per proband with high confidence (S2 Table). We developed an automated pipeline to systematically identify all candidate non-synonymous mutations in each affected individual (Fig 1). We first excluded all synonymous variants other than those occurring at canonical splice sites. This first step reduced the number of candidates to an average of 9,256 non-synonymous and canonical splice site variants per proband. We further reduced this number to 76 variants and 15 coding indels by excluding variants found in public databases; dbSNP137 [19], 1000 Genomes Project [20], NHLBI Exome Variant Server (ESP6500) [21], the Human Genetic Variation Database (HGVD: http://www.genome.med.kyoto-u.ac.jp/SnpDB). We also used our in-house whole exome or whole genome database composed of 1,257 Japanese individuals. We then excluded the variants predicted as benign/tolerant by both SIFT (http://sift.jcvi.org/www/) [22] and PolyPhen-2 (http://genetics.bwh.harvard.edu/pph2/) [23], and finally selected candidate mutations that co-segregated among affected individuals within each of the pedigrees (S2 Table). We identified 92 candidate pathogenic mutations in 88 genes in 23 out of the 35 families (65.7%), all of which were validated by Sanger sequencing. These are eleven *de novo*, five recessive (two homozygous and three compound heterozygous) and seventy-three dominant mutations (S3 Table). No gene was found to be commonly mutated among pedigrees.

### Protein-protein interaction (PPI) network analysis

**Fig 2:**
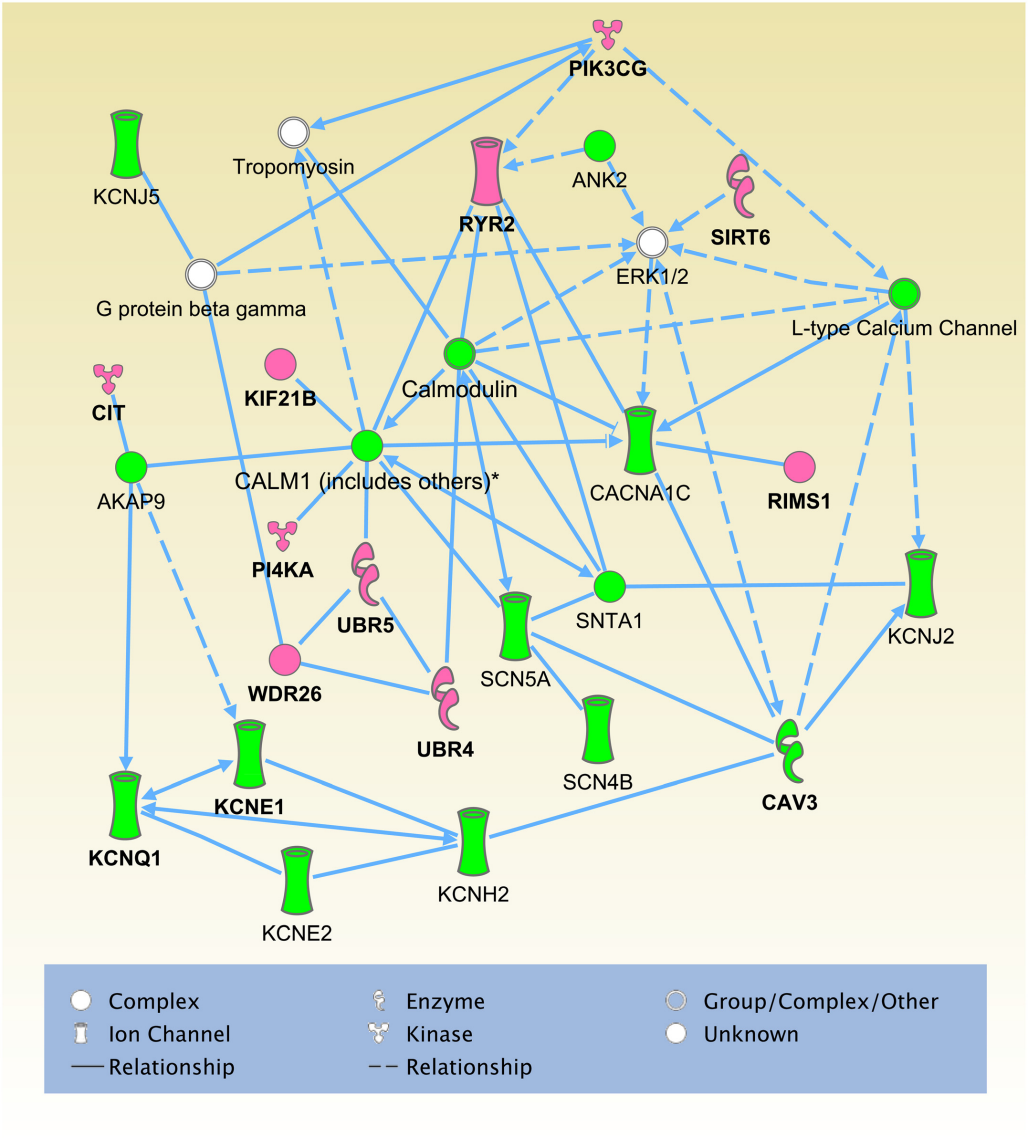
The top-scoring IPA network constructed on the basis of known genes/proteins and candidate pathogenic genes/proteins identified. The green and pink objects represent known LQTS genes and candidate pathogenic genes identified in this PPI analysis, respectively.

We applied PPI network analysis to a gene set of the 15 known genes and the 88 candidate pathogenic genes identified in this analysis, in order to elucidate any enrichment of functional units or categories. Using Ingenuity Pathways analysis software (IPA; Ingenuity Systems), we identified an interesting network, ranked top in IPA network score, composed of proteins encoded by all 15 known genes and 10 candidate pathogenic genes. Seven of the 10 pathogenic candidates were found to directly interact with at least one protein encoded by known LQTS genes (Fig 2) and contain candidate mutations that occur at evolutionarily conserved amino acids (S5 Fig) which were predicted to be damaging by SIFT [22] or PolyPhen-2 [23] analysis and to have a strong functional impact on the gene (Table 3). In addition, half of the 10 pathogenic candidates were calmodulin-interacting genes (*RYR2*, *UBR4*, *UBR5*, *PI4KA* and *KIF21B*) (Fig 2), which was statistically significant when compared to the number of molecules that directly interact with calmodulin (p = 0.042, Fisher’s exact test). We previously reported that calmodulin mutations are associated with LQTS [30]. These results suggest an important role of calmodulin and its interacting proteins in the pathogenesis of LQTS. Through PPI analysis, we could detect candidate mutations in 12 families.

**Table 3:** Potential pathogenic mutations detected in PPI analysis and Gene based Association Study (GAS) using independent samples.

ID	Gene	Model†	Transcript ID	cDNA level change	Protein level change	SIFT/PolyPhen-2*	Analysis
T02	*WDR26*	*De novo*	NM_025160.6	c.612G>T	p.L204F	T/-	PPI
T08	*RYR2*	*De novo*	NM_001035.2	c.12272C>T	p.A4091V	D/D	PPI
T12	*UBR5*	AR (CHTZ)	NM_015902.5	c.5837A>G	p.H1946R	D/P	PPI
				c.3752G>A	p.R1251H	D/B	PPI
T17	*UBR4*	*De novo*	NM_020765.2	c.6397G>A	p.A2133T	T/D	PPI
T21	*KIF21B*	*De novo*	NM_017596.2	c.3601C>T	p.R1201W	D/D	PPI
D02	*SLC2A5*	AD	NM_003039.2	c.808C>T	p.R270W	D/D	GAS
D03	*CIT*	AD	NM_001206999.1	c.5786C>A	p.S1929Y	D/D	PPI
D04	***KCNQ1***	AD	NM_000218.2	c.683+2T>G	-	-/-	PPI
D07	***CAV3***	AD	NM_033337.2	c.37A>T	p.I13F	T/B	PPI
D08	***KCNQ1***	AD	NM_000218.2	c.1032+1G>A	-	-/-	PPI
D09	***KCNE1***	AD	NM_000219.3	c.253G>A	p.D85N	D/P	PPI
D10	*SIRT6*	AD	NM_016539.2	c.742C>T	p.R248C	D/D	PPI
	*PIK3CG*	AD	NM_002649.2	c.574G>A	p.D192N	T/D	PPI
D14	*PI4KA*	AD	NM_058004.2	c.247G>A	p.D83N	D/D	PPI
	*RIMS1*	AD	NM_014989.4	c.1477G>C	p.E493Q	D/D	PPI

*D = damaging; P = probably damaging; T = tolerated; B = benign.

^†^AR: autosomal recessive (CHTZ = compound heterozygous), AD: autosomal dominant. Bold: known LQTS genes.

### Candidate gene-based association study using an independent set of case/control samples

**Fig 3:**
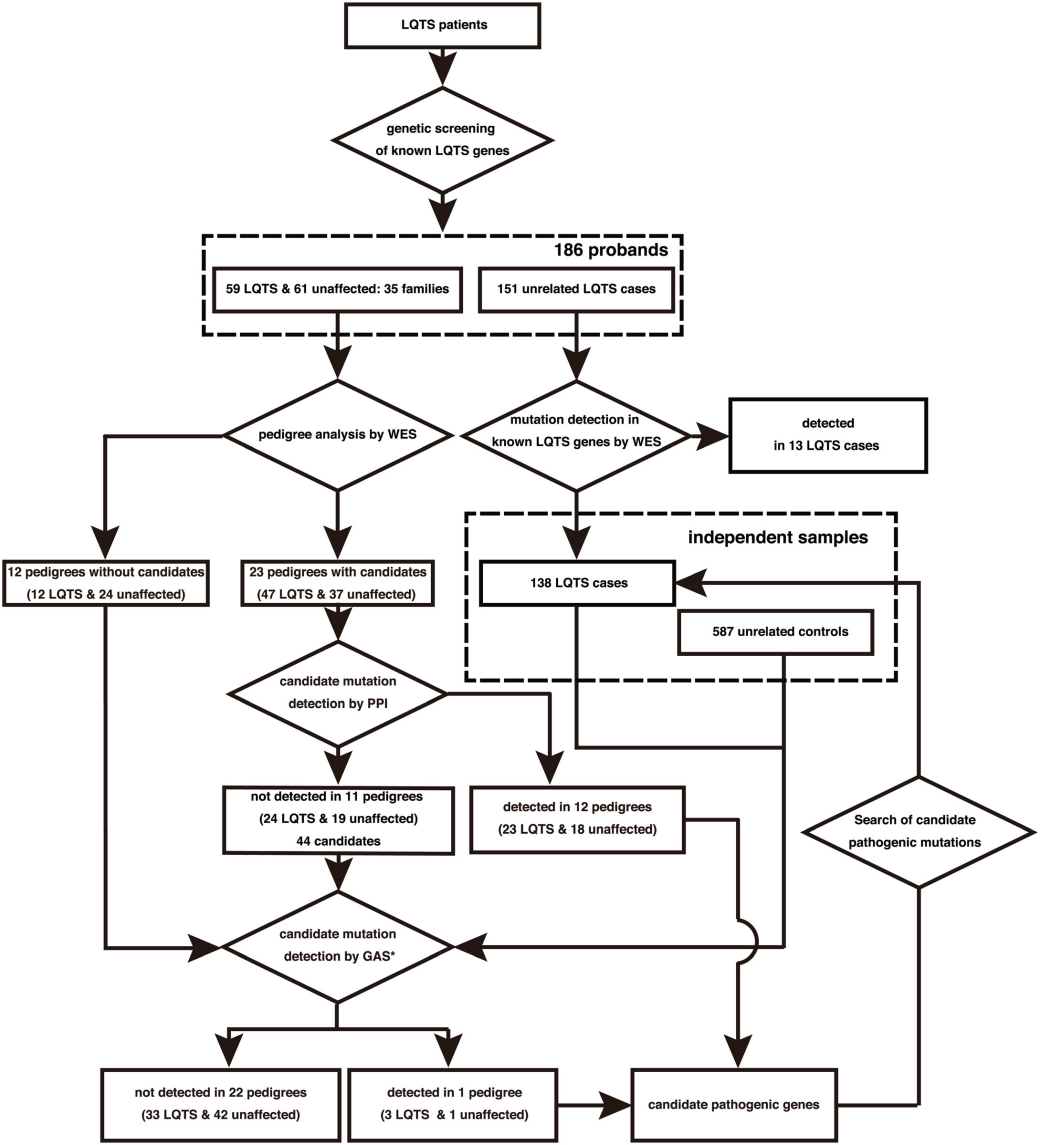
Experimental work flow for detecting candidate pathogenic mutations.

We could not identify candidate pathogenic genes supported by PPI analysis for the remaining 11 families, although 44 genes were still candidates. Therefore, we performed candidate gene-based association studies using the sequence kernel association optimal test: SKAT-O (Fig 3, see Materials and Methods) [27], in order to identify likely pathogenic genes with cumulative effects in LQTS patients from the 44 candidate genes. We used 11 probands from each of these families, 12 probands from each of the families in which no candidates were identified by pedigree analysis, and a set of 138 genetically unrelated LQTS cases and 587 controls (Fig 3). In total, 161 cases and 587 controls were examined and a significant association in the *SLC2A5* gene (also known as *GLUT5*, FDR-adjusted p-value (q-value) = 0.014, Tables 3 and 4) was found.

**Table 4:** Significant association of *SLC2A5* detected by gene-based association study.

			Case			Control			
Transcript ID	cDNA level change	Protein level change	11	12	22	11	12	22	q-value
NM_003039.2	c.888C>G	p.I296M	0	4	157	0	1	585	0.014
	c.808C>T	p.R270W	0	1	160	0	0	587	
	c.457C>G	p.L153V	0	1	159	0	1	586	

### Candidate pathogenic mutations in an independent set of unrelated LQTS cases

Investigation into the presence of possible mutations in these 11 genes in 138 genetically independent cases identified 16 candidate pathogenic mutations in 15 individuals (Table 5, candidate mutations in both *WDR26* and *RYR2* were identified in the same individual), which were non-synonymous variants and absent from in-house/public variant databases. Out of the 16 candidate mutations, 14 were calmodulin-interacting genes (87.5%, Fig 2), and 9 of these occurred at evolutionarily conserved amino acids (64.3%): four were missense variants in *RYR2*, three in *UBR4*, one in *PI4KA* and one in *KIF21B* (Table 5). Functional analysis of these mutations though evolutionarily conserved amino acid residue examination showed these mutations to be strong candidates.

**Table 5:** Candidate mutations in independent unrelated cases.

Gene	Transcript ID	cDNA level change	Protein level change	SIFT /PolyPhen-2*	Evolutionally conserved amino acid†
*RYR2*	NM_001035.2	c.497C>G	p.S166C	D/D	Conserved
*RYR2*	NM_001035.2	c.1259G>A	p.R420Q	D/D	
*RYR2*	NM_001035.2	c.1298T>C	p.L433P	D/B	Conserved
*RYR2*	NM_001035.2	c.5278C>T	p.R1760W	D/D	
*RYR2*	NM_001035.2	c.8470C>T	p.R2824W	D/D	
*RYR2*	NM_001035.2	c.11017C>T	p.R3673W	D/D	
*RYR2*	NM_001035.2	c.12438G>C	p.E4146D	D/D	Conserved
*RYR2*	NM_001035.2	c.13780A>C	p.K4594Q	D/D	Conserved
*UBR4*	NM_020765.2	c.1097A>G	p.K366R	T/P	Conserved
*UBR4*	NM_020765.2	c.1349G>T	p.R450L	D/D	Conserved
*UBR4*	NM_020765.2	c.1557G>C	p.Q519H	D/D	Conserved
*UBR5*	NM_015902.5	c.2965C>T	p.R989W	-/-	
*PI4KA*	NM_058004.2	c.738C>G	p.I246M	T/P	Conserved
*KIF21B*	NM_017596.2	c.2224G>A	p.E742K	D/P	Conserved
*CIT*	NM_001206999.1	c.5783C>T	p.A1928V	-/-	Conserved
*WDR26*	NM_001115113.2	c.59G>A	p.G20E	T/-	

*D = damaging; P = probably damaging; T = tolerated; B = benign.

^†^Conserved: evolutionally conserved amino acid in seven organisms: *Homo sapiens, Macaca mulatta, Mus musculus, Canis familiaris, Gallus gallus, Xenopus tropicalis and Danio rerio*.

Only candidate mutations in *WDR26* (c.59G>A [p.G20E]) and *RYR2* (c.11017C>T [p.R3673W]) were identified in the same individual.

Interestingly, nine (including 6 novel) mutations were identified in the *RYR2* gene, which were found in younger patients with no affected family members (Table 6). Many of the patients with the *RYR2* mutation had similar exercise-induced cardiac events (4 syncope, 2 VF, 1 cardiac arrest). This frequency was also higher and more severe compared with that in genotype-unknown LQTS, while the QTc interval was shorter in patients with the *RYR2* mutation than that with genotype-negative LQTS (439 ± 30 vs. 471 ± 50 ms; p-value = 0.01), strengthening the importance of *RYR2* in LQTS pathogenesis.

**Table 6:** Clinical background of patients with long-QT interval and *RYR2* mutation

cDNA level change	Protein level change	age	sex	Affected family members	QTc	event
c.497C>G	p.S166C	11	F	none	416	Syncope during swim, novel
c.1259G>A	p.R420Q	14	M	none	412	Syncope during swim (12 y), SD (17 y)
c.1298T>C	p.L433P	18	F	none	452	VF during exercise (17 y)
c.5278C>T	p.R1760W	16	M	none	425	Syncope during swim, novel
c.8470C>T	p.R2824W	7	M	none	439	Asympt, novel
c.11017C>T	p.R3673W	16	M	none	469	Heart failure, novel
c.12272C>T	p.A4091V	16	M	none	443	CA during exercise
c.12438G>C	p.E4146D	2	F	none	401	VF, novel
c.13780A>C	p.K4594Q	12	F	none	496	Syncope during swim (10 y), novel

## Discussion

We sequenced the exomes of 59 LQTS individuals and 61 unaffected individuals from 35 families and systematically identified candidate mutations in the affected individuals. Subsequent PPI network analysis revealed that a statistically significant proportion of pathogenic candidate molecules interacted directly with calmodulin (*RYR2* [31], *UBR4* [32], *UBR5* [33], *PI4KA* [34] and *KIF21B* [34]). Calmodulin is a primary sensor of intracellular calcium levels in eukaryotic cells, playing a key role in the proper mediation of Ca^2+^ signaling, and interacts with several known LQTS genes (*SCN5A* [35], *SNTA1* [36] and *CACNA1C* [37]), giving strength to the possibility that these candidate genes also play a pathogenic role in LQTS. In particular, *RYR2* has previously been reported as gene associated with several arrhythmic diseases, including LQTS [38], catecholaminergic polymorphic ventricular tachycardia (CPVT) [39–41], arrhythmogenic right ventricular dysplasia type 2 [42–44] and sudden infant death syndrome [45]. Along with one candidate non-synonymous mutation (c.12892G>A [p.V4298M]) in *RYR2* that has been previously reported in LQTS [38], we identified nine additional candidate mutations (Table 6), strengthening the importance of *RYR2* in LQTS pathogenesis. PPI network analysis also revealed candidate pathogenic genes that interact directly or indirectly with known LQTS genes (*RIMS1* [46], *CIT* [47], *PIK3CG* [48], *SIRT6* [49] and *WDR26* [33]), implying that these candidate genes might also cause LQTS. In particular, *RIMS1* has been reported to regulate insulin secretory machinery [50]. Since insulin infusion has been shown to cause QTc prolongation in animal models [51, 52], this gene may be more likely to play a pathogenic role in LQTS.

A candidate gene based association study also identified an additional candidate pathogenic gene, *SLC2A5*, encoding a facilitated glucose/fructose transporter that plays a fundamental role in the pathogenesis of fructose-induced hypertension [53]. Since the mechanistic link between hypertension and fatal arrhythmia is not well-characterized, the role of this gene in the pathogenesis of long QT syndrome requires further investigation.

We examined the presence of mutations in the 11 candidate pathogenic genes in the genetically independent individuals. Most of the mutations were observed in calmodulin-interacting genes or known LQTS interacting genes (15 out of 16, Table 5), and many of these occurred at evolutionarily conserved amino acid across multiple species (10 out of 15). Since amino acid substitutions at evolutionarily conserved positions could potentially lead to deleterious effects on gene functions, these mutations may play an important role in the pathogenesis of LQTS.

To our knowledge, this study is the largest whole-exome sequencing analyses for LQTS. Our analysis revealed several novel candidate pathogenic genes through PPI analysis and gene-based association study. We believe our findings will be an anchor point for finding novel pathogenesis of this disorder.

## Supporting Information

S1 Fig Pedigree data. Samples with an asterisk were subject to WES analysis and those with a question mark have unknown affected status.
(PDF)

S2 Fig Relatedness among Japanese, Han Chinese, European and African individuals. Plot of the first and the second principle components of the 749 subjects along with 45 East Asian (HapMap populations of Japanese in Tokyo: JPT), 45 Han Chinese in Beijing: CHB), 90 African (HapMap population of Yoruba in Ibadan, Nigeria: YRI), and 90 European (HapMap population of Utah, USA residents with ancestry from northern and western Europe: CEU) populations. The one outlier indicated by the arrow (case) was excluded.
(PDF)

S3 Fig A quantile-quantile (QQ) plot for association results. The genomic inflation factor *λ*_*GC*_ was 1.027.
(TIFF)

S4 Fig Coverage plots of all 120 individuals. Each line corresponds to one of the 120 individuals. On average, 92.8% of all target exons had at least 10-fold coverage.
(PDF)

S5 Fig Missense mutations observed at evolutionally conserved amino acids across seven species. Homologous sequences were aligned using CLUSTALW. We identified evolutionally conserved amino acid across seven organisms: *Homo sapiens*, *Macaca mulatta*, *Mus musculus*, *Canis familiaris*, *Gallus gallus*, *Xenopus tropicalis* and *Danio rerio*.
(PDF)

S1 Table Overview of exome-sequencing performance. † Proband.
(DOCX)

S2 Table Variants detected in each of the 35 probands. † NS: non-synonymous SNV, SP: splice-site SNV. * Confirmed candidates: candidates co-segregated in the pedigree and validated using Sanger sequencing.
(DOCX)

S3 Table Potential pathogenic mutations detected in 23 of the 35 families. † AR: autosomal recessive (HMZ = homozygous, CHTZ = compound heterozygous), AD: autosomal dominant. Bold: LQTS-susceptibility genes.
(DOCX)
