# Supplementary material for: Exome Analyses of Long QT Syndrome Reveal Candidate Pathogenic Mutations in Calmodulin-Interacting Genes
Source: PLoS One. 2015 Jul 1;10(7):e0130329. doi: 10.1371/journal.pone.0130329 (PMC4488844; doi:10.1371/journal.pone.0130329)
Supplement: S1 Fig — Samples with an asterisk were subject to WES analysis and those with a question mark have unknown affected status. (PDF) [file pone.0130329.s001.pdf]

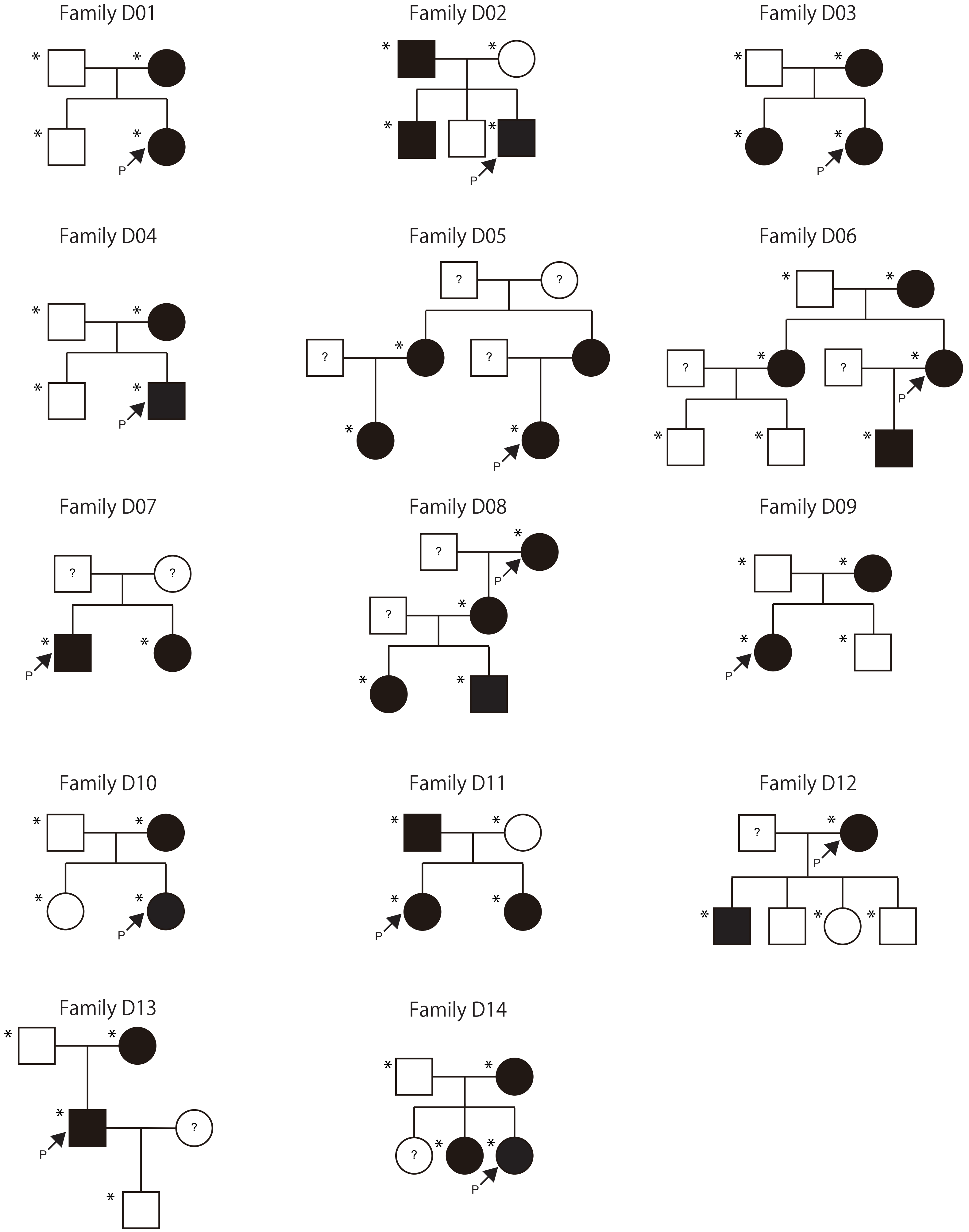
Family D01
Family D02
Family D03
Family D04
Family D05
Family D06
Family D07
Family D08
Family D09
Family D10
Family D11
Family D12
Family D13
Family D14
