# Supplementary figures and images for: Exome Analyses of Long QT Syndrome Reveal Candidate Pathogenic Mutations in Calmodulin-Interacting Genes
Source: PLoS One. 2015 Jul 1;10(7):e0130329. doi: 10.1371/journal.pone.0130329 (PMC4488844; doi:10.1371/journal.pone.0130329)

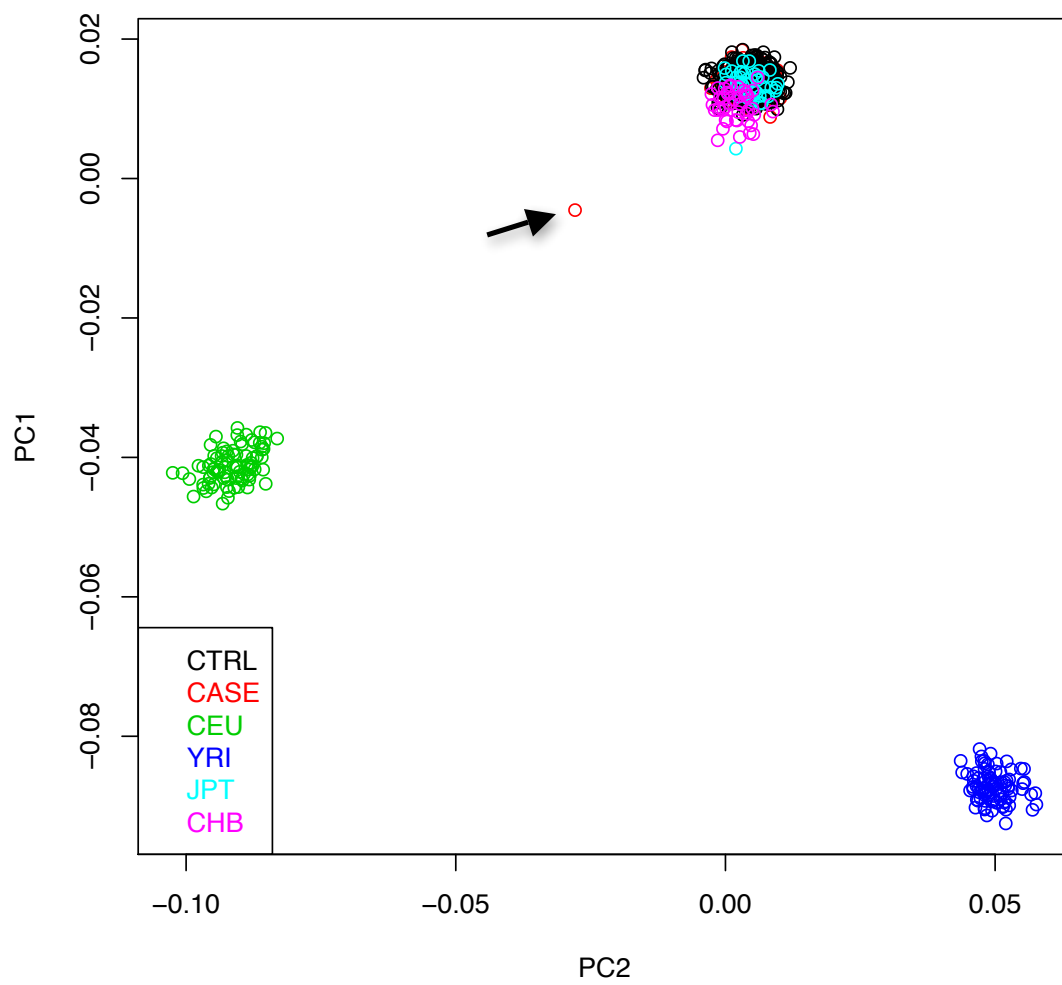

Supplement: S2 Fig — Plot of the first and the second principle components of the 749 subjects along with 45 East Asian (HapMap populations of Japanese in Tokyo: JPT), 45 Han Chinese in Beijing: CHB), 90 African (HapMap population of Yoruba in Ibadan, Nigeria: YRI), and 90 European (HapMap population of Utah, USA residents with ancestry from northern and western Europe: CEU) populations. The one outlier indicated by the arrow (case) was excluded. (PDF) [file pone.0130329.s002.pdf]

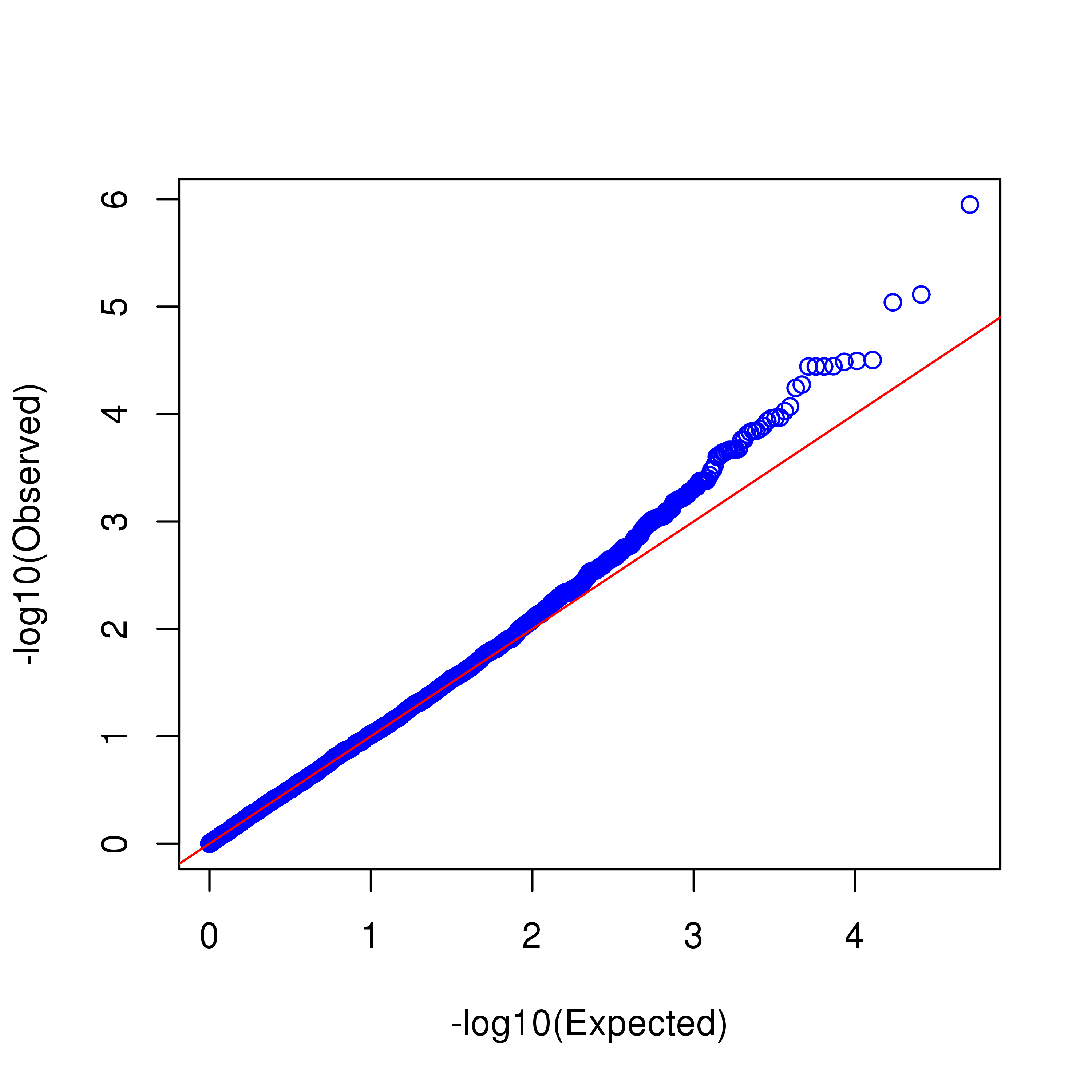

Supplement: S3 Fig — The genomic inflation factor λ GC was 1.027. (TIFF) [file pone.0130329.s003.tiff]

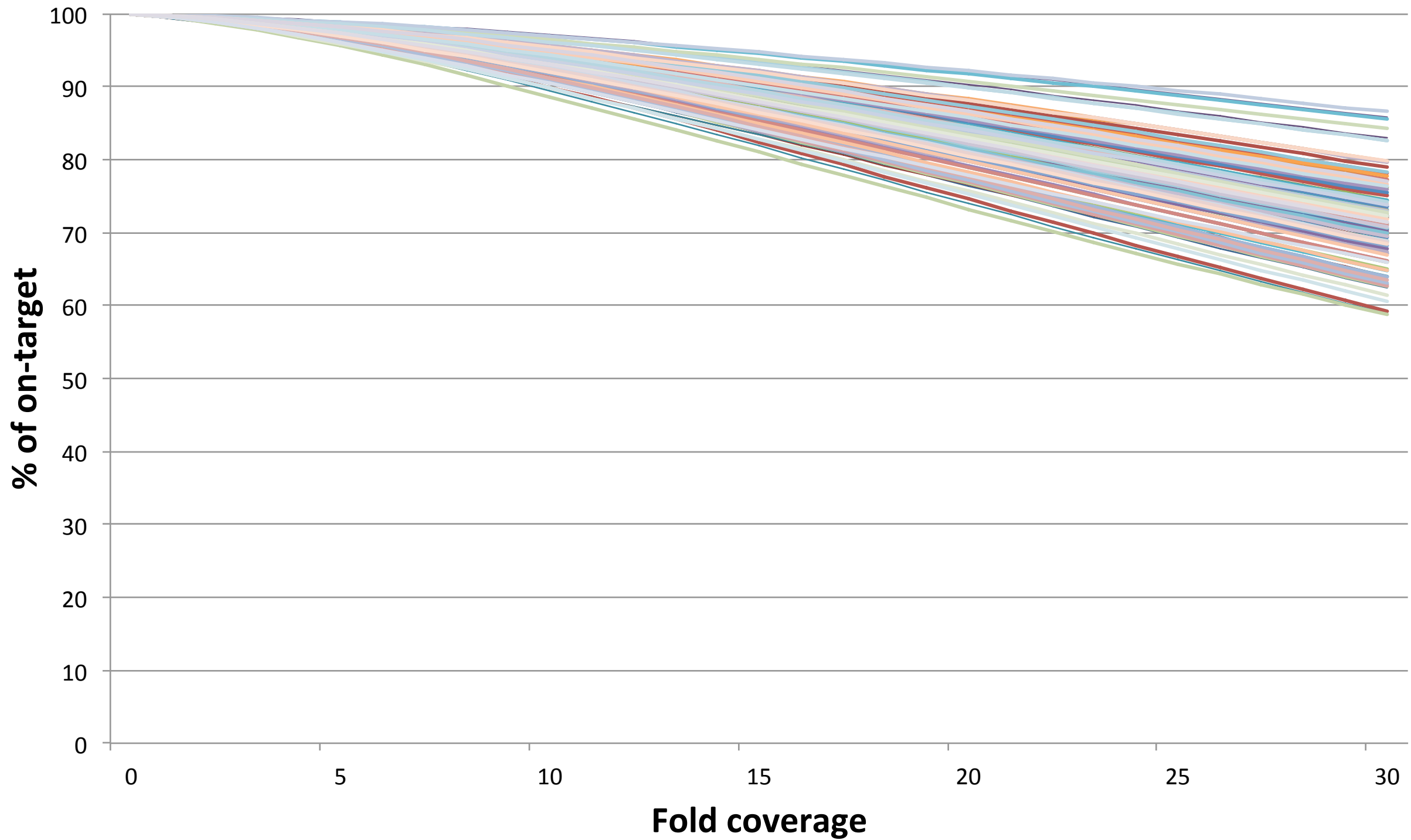

Supplement: S4 Fig — Each line corresponds to one of the 120 individuals. On average, 92.8% of all target exons had at least 10-fold coverage. (PDF) [file pone.0130329.s004.pdf]
