# Supplementary material for: Exome Analyses of Long QT Syndrome Reveal Candidate Pathogenic Mutations in Calmodulin-Interacting Genes
Source: PLoS One. 2015 Jul 1;10(7):e0130329. doi: 10.1371/journal.pone.0130329 (PMC4488844; doi:10.1371/journal.pone.0130329)
Supplement: S5 Fig — Homologous sequences were aligned using CLUSTALW. We identified evolutionally conserved amino acid across seven organisms: Homo sapiens, Macaca mulatta, Mus musculus, Canis familiaris, Gallus gallus, Xenopus tropicalis and Danio rerio. (PDF) [file pone.0130329.s005.pdf]

calmodulin-interacting genes

Gene	RYR2 (p.A4091V)	UBR4 (p.A2133T)	UBR5 (p.H1946R, p.R1251H)	PI4KA (p.D83N)	KIF21B (p.R1201W)
Homo sapiens	H E P **A** K D I	K S F **A** A T I	S L K **H** V A Y ... K S A **R** L D L	L L S **D** V A Y	L P T **R** G S T
Macaca mulatta	H E P **A** K D I	K S F **A** A T I	S L K **H** V A Y ... K S A **R** L D L	L L S **D** V A Y	L P T **R** G S T
Mus musculus	H E P **A** K D I	R S F **A** A T V	S L K **H** V A Y ... K S A **R** L D L	L L S **D** V A C	L P T **R** G S T
Canis familiaris	H E P **A** K D I	R S F **A** A T V	S L K **H** V A Y ... K S A **R** L D L	L L S **D** V A Y	L P T **R** G S T
Gallus gallus	H E P **A** K D I	K S F **A** A T I	S L K **H** V A Y ... K S A **R** L D L	L L S **D** V A Y	L P T **R** G S T
Xenopus tropicalis	Q E P **A** K D I	K S F **A** A T I	S L K **H** V A Y ... K S A **R** L D L	L L S **D** V A H	L P I **R** G N T
Danio rerio	H E P **A** K D I	K S F **A** A T V	S L K **H** V A Y ... K S A **R** L D L	L L S **D** V A Q	L P I **R** G H T

candidates directly interact with known LQTS genes

Gene	CIT (p.S1929Y)	RIMS1 (p.E493Q)	PIK3CG (p.D192N)	WDR26 (p.L204F)	SIRT6 (p.R248C)	SLC2A5 (p.R270W)
Homo sapiens	Y L A **S** S Y Q	E K V **E** T M L	A S R **D** P K L	S Q T **L** L G I	K H D **R** H A D	S R M **R** F L K
Macaca mulatta	Y L A **S** S Y Q	E K V **E** T M L	A S R **D** P R L	S Q T **L** L G I	K H D **R** H A D	S R M **R** F L K
Mus musculus	Y L A **S** S Y Q	E K A **E** S M L	A G R **D** A K L	S Q T **L** L G I	K H D **R** Q A D	S Q M **T** F L K
Canis familiaris	Y L A **S** S Y Q	E K V **E** T M L	A G R **D** P E L	S Q T **L** L G I	K H D **R** H A D	S R M **S** F L K
Gallus gallus	Y L A **S** S Y Q	E K M **E** T M L	A C R **D** P K L	F Q I **Y** L - L	- - - **-** - - -	- - - **-** - - -
Xenopus tropicalis	Y I A **S** V Y Q	E K M **E** T M L	S R R **D** A R L	- - - **-** - - -	K H - **K** H A D	P R F **T** C L N
Danio rerio	Y L A **S** S Y Q	Q K A **E** S M L	S D R **D** P N L	S - - **-** - - -	K H A **R** G Q S	S R F **T** F L N
