# Supplementary material for: Exome Analyses of Long QT Syndrome Reveal Candidate Pathogenic Mutations in Calmodulin-Interacting Genes
Source: PLoS One. 2015 Jul 1;10(7):e0130329. doi: 10.1371/journal.pone.0130329 (PMC4488844; doi:10.1371/journal.pone.0130329)
Supplement: S1 Table — † Proband. (DOCX) [file pone.0130329.s006.docx]

**S1 Table. Overview of exome-sequencing performance**

| family ID | reads sequenced (a) | reads mapped (b) | % mapping rates (b/a) | % mapping to targeted sequence | fold coverage |
| --- | --- | --- | --- | --- | --- |
| T01 |  |  |  |  |  |
| Case† | 42,691,366 | 42,070,438 | 98.55 | 69.87 | 48.08 |
| Father | 68,706,254 | 67,683,162 | 98.51 | 69.22 | 72.55 |
| Mother | 62,512,202 | 61,450,304 | 98.30 | 69.31 | 65.90 |
| T02 |  |  |  |  |  |
| Case† | 67,383,098 | 66,150,946 | 98.17 | 69.45 | 70.32 |
| Father | 56,769,692 | 55,912,420 | 98.49 | 68.44 | 62.29 |
| Mother | 50,201,164 | 49,361,444 | 98.33 | 66.10 | 54.61 |
| T03 |  |  |  |  |  |
| Case† | 50,275,528 | 49,623,134 | 98.70 | 70.92 | 56.21 |
| Father | 89,226,682 | 87,980,840 | 98.60 | 67.15 | 99.32 |
| Mother | 55,866,694 | 55,047,402 | 98.53 | 68.16 | 60.53 |
| T04 |  |  |  |  |  |
| Case† | 53,838,782 | 52,834,220 | 98.13 | 70.30 | 59.26 |
| Father | 64,637,148 | 63,713,188 | 98.57 | 68.30 | 69.81 |
| Mother | 54,551,302 | 53,699,854 | 98.44 | 68.34 | 58.98 |
| T05 |  |  |  |  |  |
| Case† | 57,864,242 | 57,242,530 | 98.93 | 68.64 | 66.77 |
| Father | 55,663,216 | 54,401,930 | 97.73 | 68.36 | 56.34 |
| Mother | 50,218,130 | 49,465,368 | 98.50 | 68.30 | 54.88 |
| T06 |  |  |  |  |  |
| Case† | 51,792,588 | 51,282,798 | 99.02 | 68.92 | 49.39 |
| Father | 59,953,296 | 58,932,898 | 98.30 | 67.07 | 64.22 |
| Mother | 66,430,654 | 65,402,856 | 98.45 | 68.04 | 69.88 |
| T07 |  |  |  |  |  |
| Case† | 59,050,478 | 58,449,748 | 98.98 | 69.34 | 53.53 |
| Father | 100,318,396 | 98,952,802 | 98.64 | 67.36 | 113.04 |
| Mother | 52,971,190 | 52,209,850 | 98.56 | 68.34 | 57.48 |
| T08 |  |  |  |  |  |
| Case† | 64,044,672 | 63,402,906 | 99.00 | 68.82 | 60.52 |
| Father | 60,499,694 | 59,664,508 | 98.62 | 68.65 | 64.47 |
| Mother | 60,531,394 | 59,623,358 | 98.50 | 68.90 | 63.84 |
| T09 |  |  |  |  |  |
| Case† | 63,127,928 | 62,537,418 | 99.06 | 68.93 | 60.88 |
| Father | 55,580,572 | 54,689,532 | 98.40 | 67.78 | 60.20 |
| Mother | 55,541,062 | 54,738,962 | 98.56 | 67.57 | 59.48 |
| T10 |  |  |  |  |  |
| Case† | 61,689,204 | 61,083,830 | 99.02 | 69.47 | 67.19 |
| Father | 85,145,918 | 83,590,620 | 98.17 | 68.53 | 83.88 |
| Mother | 60,680,796 | 59,645,512 | 98.29 | 68.16 | 66.26 |
| T11 |  |  |  |  |  |
| Case† | 95,811,076 | 94,868,146 | 99.02 | 69.11 | 86.87 |
| Father | 62,882,452 | 61,809,836 | 98.29 | 68.67 | 68.10 |
| Mother | 61,343,680 | 60,417,308 | 98.49 | 68.29 | 66.51 |
| T12 |  |  |  |  |  |
| Case† | 66,190,250 | 65,510,852 | 98.97 | 69.42 | 54.41 |
| Father | 60,276,274 | 59,580,162 | 98.85 | 68.47 | 64.59 |
| Mother | 63,710,862 | 62,924,104 | 98.77 | 69.25 | 65.99 |
| T13 |  |  |  |  |  |
| Case† | 57,336,710 | 56,827,578 | 99.11 | 68.62 | 47.55 |
| Father | 54,354,266 | 53,601,320 | 98.61 | 68.54 | 57.23 |
| Mother | 63,875,384 | 62,955,034 | 98.56 | 67.78 | 65.58 |
| T14 |  |  |  |  |  |
| Case† | 89,890,246 | 88,932,642 | 98.93 | 70.01 | 75.10 |
| Father | 74,800,364 | 73,811,752 | 98.68 | 68.08 | 79.96 |
| Mother | 63,652,606 | 62,910,022 | 98.83 | 68.69 | 69.01 |
| T15 |  |  |  |  |  |
| Case† | 56,605,156 | 55,419,986 | 97.91 | 71.45 | 59.48 |
| Father | 71,114,586 | 70,182,118 | 98.69 | 68.83 | 74.47 |
| Mother | 71,667,360 | 70,715,042 | 98.67 | 68.70 | 74.30 |
| T16 |  |  |  |  |  |
| Case† | 88,003,544 | 87,164,284 | 99.05 | 69.32 | 68.79 |
| Father | 72,365,072 | 71,465,440 | 98.76 | 68.92 | 75.57 |
| Mother | 73,145,426 | 72,087,942 | 98.55 | 67.81 | 84.31 |
| T17 |  |  |  |  |  |
| Case† | 74,128,698 | 73,493,296 | 99.14 | 69.15 | 68.38 |
| Father | 71,427,672 | 70,577,430 | 98.81 | 68.66 | 76.97 |
| Mother | 66,439,156 | 65,534,632 | 98.64 | 67.71 | 72.12 |
| T18 |  |  |  |  |  |
| Case† | 82,936,158 | 82,025,680 | 98.90 | 68.30 | 75.13 |
| Father | 66,762,816 | 65,884,854 | 98.68 | 68.08 | 71.79 |
| Mother | 64,071,196 | 63,200,474 | 98.64 | 68.44 | 69.71 |
| T19 |  |  |  |  |  |
| Case† | 75,627,192 | 74,943,510 | 99.10 | 70.49 | 86.30 |
| Father | 60,514,378 | 59,656,698 | 98.58 | 67.05 | 62.67 |
| Mother | 58,105,324 | 57,022,302 | 98.14 | 68.97 | 61.28 |
| T20 |  |  |  |  |  |
| Case† | 61,024,550 | 60,313,122 | 98.83 | 70.57 | 67.96 |
| Father | 101,060,742 | 99,017,728 | 97.98 | 68.46 | 89.30 |
| Mother | 61,016,766 | 60,316,676 | 98.85 | 69.05 | 64.21 |
| T21 |  |  |  |  |  |
| Case† | 57,172,202 | 56,587,196 | 98.98 | 68.84 | 66.22 |
| Father | 60,345,562 | 59,453,598 | 98.52 | 68.44 | 63.32 |
| Mother | 62,452,038 | 61,522,866 | 98.51 | 68.03 | 65.95 |
| D01 |  |  |  |  |  |
| Case 1† | 52,792,512 | 52,087,006 | 98.66 | 70.20 | 58.56 |
| Case 2 | 85,693,534 | 83,902,142 | 97.91 | 67.55 | 93.82 |
| Control 1 | 81,728,124 | 80,073,216 | 97.98 | 69.04 | 83.99 |
| Control 2 | 65,591,594 | 64,472,036 | 98.29 | 69.54 | 70.10 |
| D02 |  |  |  |  |  |
| Case 1† | 53,214,388 | 52,468,088 | 98.60 | 71.28 | 56.92 |
| Case 2 | 53,061,180 | 52,150,400 | 98.28 | 69.42 | 57.35 |
| Case 3 | 76,927,478 | 75,727,658 | 98.44 | 67.51 | 86.70 |
| Control 1 | 102,624,158 | 100,959,572 | 98.38 | 67.82 | 114.93 |
| D03 |  |  |  |  |  |
| Case 1† | 60,953,046 | 59,934,282 | 98.33 | 69.39 | 64.41 |
| Case 2 | 74,619,620 | 73,472,302 | 98.46 | 68.14 | 77.45 |
| Case 3 | 57,484,248 | 56,293,500 | 97.93 | 68.13 | 58.17 |
| Control 1 | 63,070,634 | 62,167,422 | 98.57 | 68.61 | 65.03 |
| D04 |  |  |  |  |  |
| Case 1† | 56,248,142 | 55,662,260 | 98.96 | 68.72 | 65.27 |
| Case 2 | 54,410,522 | 53,624,368 | 98.56 | 67.78 | 59.89 |
| Control 1 | 66,443,734 | 65,272,452 | 98.24 | 68.34 | 70.85 |
| Control 2 | 60,252,168 | 59,344,100 | 98.49 | 67.90 | 65.24 |
| D05 |  |  |  |  |  |
| Case 1† | 64,870,366 | 64,028,272 | 98.70 | 69.50 | 53.23 |
| Case 2 | 48,926,672 | 48,194,834 | 98.50 | 68.51 | 54.12 |
| Case 3 | 59,123,868 | 58,201,926 | 98.44 | 68.82 | 62.54 |
| Case 4 | 73,204,080 | 72,204,422 | 98.63 | 67.13 | 82.85 |
| D06 |  |  |  |  |  |
| Case 1† | 65,077,300 | 64,353,508 | 98.89 | 69.42 | 55.65 |
| Case 2 | 52,740,372 | 51,984,094 | 98.57 | 68.83 | 56.30 |
| Case 3 | 74,673,954 | 70,878,396 | 94.92 | 66.21 | 72.04 |
| Case 4 | 62,611,380 | 60,968,822 | 97.38 | 68.27 | 65.41 |
| Control 1 | 52,091,942 | 50,695,644 | 97.32 | 68.21 | 53.82 |
| Control 2 | 53,235,474 | 51,706,206 | 97.13 | 67.73 | 52.34 |
| Control 3 | 49,073,426 | 47,539,210 | 96.87 | 68.99 | 51.84 |
| D07 |  |  |  |  |  |
| Case 1† | 64,431,506 | 63,639,878 | 98.77 | 68.76 | 53.52 |
| Case 2 | 66,009,064 | 65,017,748 | 98.50 | 67.05 | 70.43 |
| D08 |  |  |  |  |  |
| Case 1† | 98,301,878 | 97,210,532 | 98.89 | 68.85 | 88.91 |
| Case 2 | 67,829,466 | 66,704,982 | 98.34 | 68.01 | 70.30 |
| Case 3 | 101,948,926 | 100,511,850 | 98.59 | 66.70 | 98.02 |
| Case 4 | 55,282,634 | 54,652,194 | 98.86 | 68.01 | 58.98 |
| D09 |  |  |  |  |  |
| Case 1† | 108,327,692 | 104,892,276 | 96.83 | 69.55 | 73.27 |
| Case 2 | 72,049,374 | 71,008,072 | 98.55 | 68.44 | 73.60 |
| Control 1 | 64,206,928 | 63,260,140 | 98.53 | 68.90 | 66.40 |
| Control 2 | 110,184,838 | 108,537,310 | 98.50 | 68.21 | 108.93 |
| D10 |  |  |  |  |  |
| Case 1† | 90,454,080 | 89,610,260 | 99.07 | 68.81 | 78.56 |
| Case 2 | 132,951,252 | 131,009,640 | 98.54 | 68.48 | 123.31 |
| Control 1 | 66,047,242 | 65,011,310 | 98.43 | 67.80 | 68.62 |
| Control 2 | 62,672,420 | 61,933,368 | 98.82 | 68.41 | 66.87 |
| D11 |  |  |  |  |  |
| Case 1† | 58,768,758 | 58,219,118 | 99.06 | 69.91 | 67.51 |
| Case 2 | 65,355,468 | 64,322,660 | 98.42 | 67.77 | 70.21 |
| Case 3 | 104,995,540 | 103,363,124 | 98.45 | 67.98 | 99.30 |
| Control 1 | 68,187,990 | 67,138,506 | 98.46 | 68.39 | 74.11 |
| D12 |  |  |  |  |  |
| Case 1† | 72,055,184 | 71,410,450 | 99.11 | 70.26 | 82.41 |
| Case 2 | 104,392,576 | 102,310,170 | 98.01 | 68.56 | 86.46 |
| Control 1 | 59,616,658 | 58,916,004 | 98.82 | 67.63 | 60.67 |
| Control 2 | 57,678,050 | 56,939,438 | 98.72 | 68.56 | 60.25 |
| D13 |  |  |  |  |  |
| Case 1† | 44,980,210 | 44,498,546 | 98.93 | 70.69 | 52.07 |
| Case 2 | 73,711,240 | 72,499,762 | 98.36 | 67.43 | 81.54 |
| Control 1 | 48,944,232 | 48,177,358 | 98.43 | 69.25 | 49.61 |
| Control 2 | 65,280,520 | 64,312,286 | 98.52 | 68.37 | 60.56 |
| D14 |  |  |  |  |  |
| Case 1† | 58,412,154 | 57,660,048 | 98.71 | 69.91 | 66.40 |
| Case 2 | 68,266,410 | 67,299,746 | 98.58 | 68.87 | 72.99 |
| Case 3 | 63,117,068 | 62,271,366 | 98.66 | 68.62 | 68.51 |
| Control 1 | 63,383,070 | 62,536,736 | 98.66 | 68.23 | 67.78 |
| Over all 120 individuals | | | | | |
| Avg. | 67,142,826 | 66,131,303 | 98.50 | 68.63 | 68.06 |
| Min. | 42,691,366 | 42,070,438 | 94.92 | 66.10 | 47.55 |
| Max. | 132,951,252 | 131,009,640 | 99.14 | 71.45 | 123.31 |

† Proband
