# Supplementary material for: Exome Analyses of Long QT Syndrome Reveal Candidate Pathogenic Mutations in Calmodulin-Interacting Genes
Source: PLoS One. 2015 Jul 1;10(7):e0130329. doi: 10.1371/journal.pone.0130329 (PMC4488844; doi:10.1371/journal.pone.0130329)
Supplement: S2 Table — † NS: non-synonymous SNV, SP: splice-site SNV. * Confirmed candidates: candidates co-segregated in the pedigree and validated using Sanger sequencing. (DOCX) [file pone.0130329.s007.docx]

**S2 Table.** **Variants detected in each of the 35 probands**

| pedigree# | High-confidence variant calls: coding SNVs | After exclusion of synonymous variants: NS+SP† (indels) | After exclusion of known variants: NS+SP† (indels) | Confirmed candidates*: NS+SP † (indels) |
| --- | --- | --- | --- | --- |
| T01 | 18,608 | 8,810 (479) | 59 (15) | 0 (0) |
| T02 | 19,476 | 9,253 (526) | 68 (13) | 2 (0) |
| T03 | 19,050 | 8,968 (510) | 65 (14) | 1 (0) |
| T04 | 19,125 | 9,118 (471) | 67 (17) | 0 (0) |
| T05 | 19,663 | 9,325 (497) | 81 (6) | 0 (0) |
| T06 | 18,839 | 8,987 (488) | 74 (13) | 0 (0) |
| T07 | 19,209 | 9,148 (520) | 85 (20) | 0 (0) |
| T08 | 19,727 | 9,311 (530) | 77 (12) | 3 (0) |
| T09 | 18,952 | 8,957 (503) | 83 (9) | 4 (0) |
| T10 | 19,647 | 9,315 (506) | 82 (7) | 2 (0) |
| T11 | 20,426 | 9,657 (553) | 73 (11) | 0 (0) |
| T12 | 19,202 | 9,142 (506) | 82 (12) | 2 (0) |
| T13 | 18,888 | 8,994 (499) | 65 (11) | 0 (0) |
| T14 | 20,043 | 9,537 (521) | 98 (6) | 0 (0) |
| T15 | 19,274 | 9,173 (485) | 63 (17) | 0 (0) |
| T16 | 19,914 | 9,487 (526) | 63 (10) | 0 (0) |
| T17 | 19,806 | 9,383 (535) | 96 (10) | 3 (0) |
| T18 | 19,732 | 9,386 (552) | 102 (18) | 0 (1) |
| T19 | 20,080 | 9,564 (519) | 85 (5) | 0 (0) |
| T20 | 19,254 | 9,159 (526) | 76 (16) | 0 (0) |
| T21 | 19,681 | 9,252 (535) | 72 (14) | 1 (0) |
| D01 | 19,389 | 9,213 (488) | 79 (18) | 9 (0) |
| D02 | 19,376 | 9,266 (501) | 68 (9) | 4 (0) |
| D03 | 19,442 | 9,164 (495) | 87 (14) | 10 (0) |
| D04 | 19,460 | 9,205 (499) | 62 (16) | 1 (0) |
| D05 | 19,115 | 9,060 (508) | 66 (34) | 3 (0) |
| D06 | 19,314 | 9,167 (482) | 74 (14) | 1 (0) |
| D07 | 19,282 | 9,198 (515) | 73 (35) | 1 (0) |
| D08 | 20,360 | 9,665 (558) | 75 (18) | 1 (0) |
| D09 | 20,081 | 9,464 (595) | 76 (66) | 1 (0) |
| D10 | 20,243 | 9,591 (543) | 73 (10) | 9 (0) |
| D11 | 19,802 | 9,489 (539) | 68 (15) | 11 (0) |
| D12 | 19,942 | 9,386 (522) | 77 (8) | 3 (0) |
| D13 | 19,192 | 9,209 (505) | 75 (14) | 7 (0) |
| D14 | 19,092 | 8,954 (507) | 72 (12) | 12 (0) |
| Avg. | 19,505 | 9,256 (516) | 76 (15) | 2.6 (0.03) |

† NS: non-synonymous SNV, SP: splice-site SNV

* Confirmed candidates: candidates co-segregated in the pedigree and validated using Sanger sequencing
