# Supplementary material for: Exome Analyses of Long QT Syndrome Reveal Candidate Pathogenic Mutations in Calmodulin-Interacting Genes
Source: PLoS One. 2015 Jul 1;10(7):e0130329. doi: 10.1371/journal.pone.0130329 (PMC4488844; doi:10.1371/journal.pone.0130329)
Supplement: S3 Table — † AR: autosomal recessive (HMZ = homozygous, CHTZ = compound heterozygous), AD: autosomal dominant. Bold: LQTS-susceptibility genes. (DOCX) [file pone.0130329.s008.docx]

**S3 Table. Potential pathogenic mutations detected in 23 of the 35 families**

| Pedigree # | Gene | Inherited from† | Transcript ID | cDNA level change | Protein level change |
| --- | --- | --- | --- | --- | --- |
| T02 | *WDR26* | *De novo* | NM_025160.6 | c.612G>T | p.L204F |
|  | *YME1L1* | *De novo* | NM_014263.2 | c.859G>A | p.V287M |
| T03 | *ATL3* | *De novo* | NM_015459.3 | c.955C>T | p.R319W |
| T08 | *RYR2* | *De novo* | NM_001035.2 | c.12272C>T | p.A4091V |
|  | *TCTN3* | *De novo* | NM_015631.5 | c.152C>A | p.S51X |
|  | *TOP2A* | *De novo* | NM_001067.3 | c.263T>C | p.I88T |
| T09 | *KIF11* | AR (HMZ) | NM_004523.3 | c.205G>A | p.D69N |
|  | *PROKR1* | AR (HMZ) | NM_138964.2 | c.689A>C | p.K230T |
|  | *PZP* | AR (CHTZ) | NM_002864.2 | c.3325G>A | p.A1109T |
|  |  |  |  | c.1504G>A | p.V502I |
| T10 | *CCDC168* | AR (CHTZ) | NM_001146197.1 | c.9652A>G | p.M3218V |
|  |  |  |  | c.3838G>A | p.E1280K |
| T12 | *UBR5* | AR (CHTZ) | NM_015902.5 | c.5837A>G | p.H1946R |
|  |  |  |  | c.3752G>A | p.R1251H |
| T17 | *UBR4* | *De novo* | NM_020765.2 | c.6397G>A | p.A2133T |
|  | *TRHDE* | *De novo* | NM_013381.2 | c.2047A>T | p.I683F |
|  | *ZNF174* | *De novo* | NM_003450.2 | c.1204C>T | p.R402X |
| T18 | *GPATCH2* | *De novo* | NM_018040.2 | c.1526delG | p.G509fs |
| T21 | *KIF21B* | *De novo* | NM_017596.2 | c.3601C>T | p.R1201W |
| D01 | *NET1* | AD | NM_001047160.1 | c.583C>T | p.L195F |
|  | *AKAP8* | AD | NM_005858.3 | c.787G>C | p.G263R |
|  | *ARVCF* | AD | NM_001670.2 | c.1792G>A | p.G598R |
|  | *PRSS12* | AD | NM_003619.3 | c.1516G>A | p.E506K |
|  | *ELMOD2* | AD | NM_153702.3 | c.674A>G | p.K225R |
|  | *HK3* | AD | NM_002115.2 | c.2035G>A | p.E679K |
|  | *MDN1* | AD | NM_014611.1 | c.9722T>C | p.F3241S |
|  | *INTS8* | AD | NM_017864.2 | c.484A>C | p.K162Q |
|  | *SYK* | AD | NM_001135052.2 | c.154G>T | p.A52S |
| D02 | *SLC2A5* | AD | NM_003039.2 | c.808C>T | p.R270W |
|  | *LRBA* | AD | NM_006726.3 | c.6157G>A | p.E2053K |
|  | *NLRX1* | AD | NM_024618.2 | c.2252G>A | p.R751H |
|  | *FGF2* | AD | NM_002006.4 | c.326G>A | p.G109D |
| D03 | *SLC6A17* | AD | NM_001010898.2 | c.2021G>A | p.R674H |
|  | *CIT* | AD | NM_001206999.1 | c.5786C>A | p.S1929Y |
|  | *WDR25* | AD | NM_001161476.1 | c.840C>A | p.D280E |
|  | *UBR7* | AD | NM_175748.3 | c.293G>A | p.R98H |
|  | *CD276* | AD | NM_025240.2 | c.338G>A | p.R113H |
|  | *TNFRSF6B* | AD | NM_003823.3 | c.649G>A | p.D217N |
|  | *STK32B* | AD | NM_018401.1 | c.487A>G | p.T163A |
|  | *TDRD6* | AD | NM_001010870.2 | c.4720T>G | p.C1574G |
|  | *PRSS57* | AD | NM_214710.3 | c.92G>T | p.G31V |
|  | *SHANK3* | AD | NM_001080420.1 | c.2191G>A | p.D731N |
| D04 | ***KCNQ1*** | AD | NM_000218.2 | c.683+2T>G | - |
| D05 | *ARHGAP22* | AD | NM_021226.2 | c.14A>C | p.K5T |
|  | *MYLK4* | AD | NM_001012418.3 | c.451G>A | p.E151K |
|  | *SNAPC5* | AD | NM_006049.2 | c.223A>G | p.T75A |
| D06 | *SND1* | AD | NM_014390.2 | c.1871A>C | p.E624A |
| D07 | ***CAV3*** | AD | NM_033337.2 | c.37A>T | p.I13F |
| D08 | ***KCNQ1*** | AD | NM_000218.2 | c.1032+1G>A | - |
| D09 | ***KCNE1*** | AD | NM_000219.3 | c.253G>A | p.D85N |
| D10 | *MESDC1* | AD | NM_022566.2 | c.845A>G | p.K282R |
|  | *CTRL* | AD | NM_001907.2 | c.217G>A | p.A73T |
|  | *SIRT6* | AD | NM_016539.2 | c.742C>T | p.R248C |
|  | *POLRMT* | AD | NM_005035.3 | c.2698G>A | p.E900K |
|  | *LRRC8E* | AD | NM_025061.3 | c.1777G>A | p.G593R |
|  | *ZNF341* | AD | NM_032819.3 | c.2143C>T | p.R715C |
|  | *ANKRD31* | AD | NM_001164443.1 | c.743G>A | p.R248H |
|  | *PIK3CG* | AD | NM_002649.2 | c.574G>A | p.D192N |
|  | *SNAPC4* | AD | NM_003086.2 | c.3935G>A | p.R1312Q |
| D11 | *HKDC1* | AD | NM_025130.3 | c.1001T>A | p.I334N |
|  | *RALGAPA1* | AD | NM_014990.1 | c.5996G>C | p.R1999P |
|  | *NLRP13* | AD | NM_176810.2 | c.190G>A | p.D64N |
|  | *SDC1* | AD | NM_001006946.1 | c.188C>T | p.S63F |
|  | *REM1* | AD | NM_014012.4 | c.577G>A | p.V193M |
|  | *PLCB4* | AD | NM_001172646.1 | c.448C>T | p.H150Y |
|  | *CELSR1* | AD | NM_014246.1 | c.3406G>A | p.D1136N |
|  | *NOV* | AD | NM_002514.3 | c.605C>G | p.S202X |
|  | *FSIP2* | AD | NM_173651.2 | c.4210G>A | p.E1404K |
|  | *VSX1* | AD | NM_014588.4 | c.310G>C | p.A104P |
|  | *ARL13B* | AD | NM_001174150.1 | c.379G>T | p.V127L |
| D12 | *NRIP1* | AD | NM_003489.3 | c.640A>G | p.R214G |
|  | *ABCF1* | AD | NM_001025091.1 | c.2275C>T | p.R759W |
|  | *GUF1* | AD | NM_021927.2 | c.655G>T | p.D219Y |
| D13 | *NR5A2* | AD | NM_205860.1 | c.1052A>T | p.Q351L |
|  | *MKI67* | AD | NM_002417.4 | c.7106T>C | p.V2369A |
|  | *BAIAP3* | AD | NM_001199097.1 | c.2563G>C | p.A855P |
|  | *TGFBRAP1* | AD | NM_004257.4 | c.642G>C | p.K214N |
|  | *DIP2A* | AD | NM_206889.2 | c.2654G>A | p.R885Q |
|  | *USP19* | AD | NM_001199161.1 | c.1134G>T | p.E378D |
|  | *ZNF862* | AD | NM_001099220.1 | c.637T>G | p.W213G |
| D14 | *MYBPHL* | AD | NM_001010985.2 | c.475G>A | p.G159S |
|  | *DNA2* | AD | NM_001080449.2 | c.811G>A | p.G271R |
|  | *PKD1L2* | AD | NM_052892.3 | c.1089C>A | p.C363X |
|  | *PTOV1* | AD | NM_017432.3 | c.635A>T | p.K212M |
|  | *HNRNPM* | AD | NM_031203.2 | c.76G>A | p.G26S |
|  | *PI4KA* | AD | NM_058004.2 | c.247G>A | p.D83N |
|  | *TRMU* | AD | NM_018006.4 | c.1007A>C | p.Q336P |
|  | *SIDT1* | AD | NM_017699.2 | c.2194A>G | p.K732E |
|  | *WWC2* | AD | NM_024949.5 | c.3260T>C | p.L1087P |
|  | *ERAP1* | AD | NM_016442.3 | c.955A>G | p.M319V |
|  | *RIMS1* | AD | NM_014989.4 | c.1477G>C | p.E493Q |
|  | *UPP1* | AD | NM_181597.1 | c.280C>T | p.R94C |

† AR: autosomal recessive (HMZ= homozygous, CHTZ= compound heterozygous), AD: autosomal dominant. Bold: LQTS-susceptibility genes.
